# Detection of sleep apnea from single-channel electroencephalogram (EEG) using an explainable convolutional neural network (CNN)

**DOI:** 10.1371/journal.pone.0272167

**Published:** 2022-09-13

**Authors:** Lachlan D. Barnes, Kevin Lee, Andreas W. Kempa-Liehr, Luke E. Hallum

**Affiliations:** 1 Department of Mechanical and Mechatronics Engineering, University of Auckland, Auckland, New Zealand; 2 Department of Engineering Science, University of Auckland, Auckland, New Zealand; Universiti Tunku Abdul Rahman, MALAYSIA

## Abstract

Sleep apnea (SA) is a common disorder involving the cessation of breathing during sleep. It can cause daytime hypersomnia, accidents, and, if allowed to progress, serious, chronic conditions. Continuous positive airway pressure is an effective SA treatment. However, long waitlists impede timely diagnosis; overnight sleep studies involve trained technicians scoring a polysomnograph, which comprises multiple physiological signals including multi-channel electroencephalography (EEG). Therefore, it is important to develop simplified and automated approaches to detect SA. In the present study, we have developed an explainable convolutional neural network (CNN) to detect SA events from single-channel EEG recordings which generalizes across subjects. The network architecture consisted of three convolutional layers. We tuned hyperparameters using the Hyperband algorithm, optimized parameters using Adam, and quantified network performance with subjectwise 10-fold cross-validation. Our CNN performed with an accuracy of 69.9%, and a Matthews correlation coefficient (MCC) of 0.38. To explain the mechanisms of our trained network, we used critical-band masking (CBM): after training, we added bandlimited noise to test recordings; we parametrically varied the noise band center frequency and noise intensity, quantifying the deleterious effect on performance. We reconciled the effects of CBM with lesioning, wherein we zeroed the trained network’s 1st-layer filter kernels in turn, quantifying the deleterious effect on performance. These analyses indicated that the network learned frequency-band information consistent with known SA biomarkers, specifically, delta and beta band activity. Our results indicate single-channel EEG may have clinical potential for SA diagnosis.

## Introduction

Sleep apnea (SA) is a progressive disease which involves repeated episodes of apnea and/or hypopnea during sleep. It afflicts between 2% and 7% of the general population [1, 2] and causes fragmented sleep. Sufferers often experience daytime hypersomnia, cognitive dysfunction that accompanies sleepiness, and an increased risk of workplace and motor vehicle accidents [3]. If allowed to progress, SA is associated with a range of serious, chronic conditions, including cardiovascular and cerebrovascular disease, and diabetes [1, 2, 4, 5]. Continuous positive airway pressure (CPAP)—the gold standard SA treatment—is highly effective [4]. However, there are barriers to SA detection, diagnosis, and treatment. The primary clinical tool for SA detection and diagnosis is overnight polysomnography (PSG), which involves sleep studies and manual scoring of recorded physiological signals by trained healthcare professionals [6]. PSG is instrumentation-intensive—it typically involves monitoring nasal or oral airflow; thoracic and/or abdominal movement; snoring; oxygen saturation; multi-channel electroencephalogram (EEG); electrooculogram (EOG); electrocardiogram (ECG); and, electromyogram (EMG)—and therefore, may interfere with standard patterns of sleep [7]. Overnight PSG is typically followed by manually titrated CPAP therapy [1].

Demand for overnight PSG exceeds supply; wait times for those requiring screening and diagnosis can range from 2 to 60 months in selected developed countries [8], and it is estimated that a large proportion of SA sufferers remain undiagnosed [9]. Tools to aid detection, diagnosis, and treatment of SA are therefore a worthy pursuit, especially those involving simplified instrumentation and automation, with potential for use in the home as well as the clinic [10–12]. Automated systems making use of explainable machine learning [13] could, potentially, be used to expedite and augment diagnostic and treatment decisions; in general, explainable systems are those wherein mechanisms of detection and/or classification are made available to clinicians. Automated detection systems have been explored [14] using various modalities such as ECG [15, 16], oxygen saturation [17], or airflow [18–20]. EEG contains a rich variety of physiological signals enabling objective measurement of sleep stage, as well as various sleep disorders (e.g., narcolepsy [21]). Hence EEG-based detection may have far-reaching applications in simplified at-home sleep tests and may offer a constructive perspective to present sleep monitoring modalities. It is also noteworthy that substantial progress has been made to improve the instrumentation and applicability of EEG for wearable devices [12] and the production of consumer level products [22] in pursuit of better health monitoring and sleep assessment.

The traditional approach to SA detection from EEG involves computation of features (e.g., energy and energy variance [23]) within predefined frequency bands [23, 24]. Features are concatenated to form a high-dimensional feature vector for use in classification. Convolutional neural networks (CNNs) are a form of artificial neural network, loosely inspired by hierarchical, computational models of visual processing in the cerebral cortex (review by LeCun et al. [25]). CNNs use convolution as a form of shift-invariant feature extraction, and learn, through training, to extract salient features from time series signals (or images) that are useful for classification. Recently, CNNs have demonstrated proficiency for the classification of images [26] and signals across a range of domains, including multi-channel EEG [27]. In contrast to traditional approaches, the CNN we develop here requires no postulation of features and frequency bands at the outset, meaning that features not traditionally associated with SA could be learned during the training procedure. Beside CNNs, there are several other state-of-the-art machine learning algorithms such as residual neural networks [20], and transformer architectures [28]. Although such architectures may offer superior performance in some domains compared to CNNs, it can come with the cost of explainability; explainability is a requirement for healthcare applications [29]. Furthermore, our CNN uses single-channel EEG; we are interested in the feasibility of a wearable sleep assessment device, analogous to recent single-lead electrocardiographic adhesive patches for detecting cardiac arrhythmia [30].

We hypothesized, first, that there is information in single-channel EEG enabling reliable subjectwise detection of SA by a CNN; by “subjectwise”, we mean a CNN trained using data collected from a cohort of subjects, 1 through N, should generalize to detect SA in a previously unseen subject, N+1. Second, we hypothesized that knowledge of sleep stage (e.g., rapid-eye-movement sleep) should improve this SA detection. This hypothesis is reasonable because both SA as well as sleep stage are accompanied by characteristic alterations of EEG, and SA is associated with sleep stage (a point we elaborate in the Discussion). Third, we hypothesized that the network features enabling SA detection should be consistent with known SA biomarkers. To test these hypotheses, we trained a CNN to detect SA using single-channel EEG. To explain the trained CNN’s mechanisms, we used two visualization techniques—critical-band masking [31, 32] (wherein band-limited noise was added to signals used to test the trained network) and filter lesioning [33] (wherein 1st-layer filter kernels comprising the trained network were zeroed in turn, and the effect on performance was quantified).

## Materials and methods

### Datasets

We used three datasets. Our CNN was trained and tested using data drawn from Sleep Health Heart Study (SHHS) Visit 2, which contains overnight EEG recordings from 2,650 patients sampled at either 125 Hz or 128 Hz [34, 35]. This dataset is publicly available via the National Sleep Research Resource which provides access to large collections of de-identified physiological signals for research purposes (https://sleepdata.org) [34]. We performed further testing of our trained CNN using data from the St. Vincent’s University Hospital / University College Dublin Sleep Apnea Database [36], consisting of recordings from 25 participants sampled at 128 Hz, and the MIT-BIH Polysomnographic Database [36, 37], consisting of usable recordings from 16 patients sampled at 250 Hz. These latter two datasets are publicly available via PhysioNet which provides access to a collection of de-identified physiological signals for research purposes (https://www.physionet.org) [36]. The University of Auckland Human Participants Ethics Committee waived the requirement for any further approval to use these datasets in this study.

Sleep apnea annotation procedures differed considerably between datasets. These differences presumably hindered our ability to develop a single CNN using the SHHS data set which generalised to both the UCD and MIT data sets (see Discussion). The SHHS dataset scored sleep apneas using internally developed rules [35], and the scoring procedure involved multiple stages of both manual and computer-automated annotation. Few details regarding the UCD and MIT scoring were available. Furthermore, MIT data were pre-segmented (30-second segments) and pre-labelled, whereas SHHS and UCD data were annotated at finer temporal resolution.

**Fig 1:**
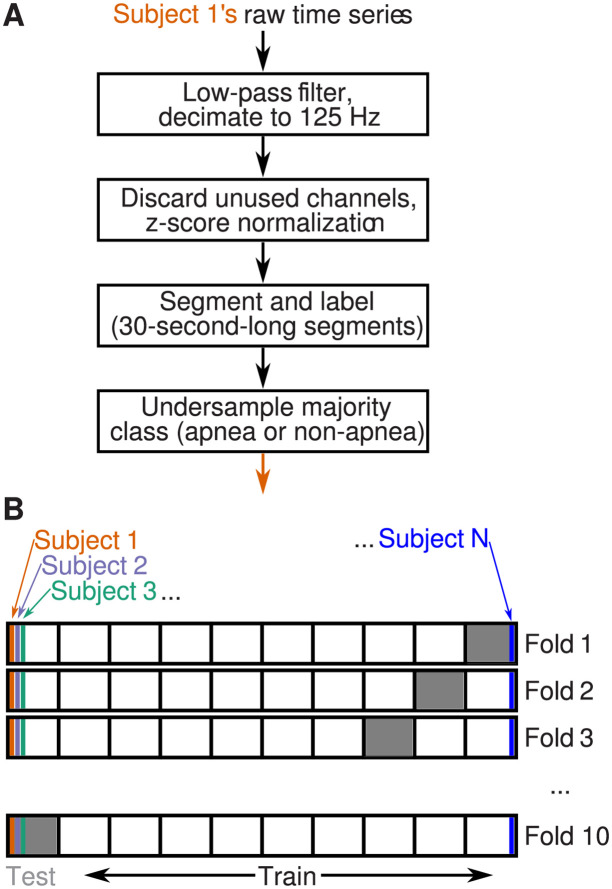
Data processing and subjectwise 10-fold cross-validation. (A) We used recordings from the SHHS dataset [34, 35]. For each subject, we low-pass filtered, downsampled, normalised, segmented, labelled, and undersampled recordings as indicated. (B) On each fold of our 10-fold cross-validation procedure, any subject’s recordings appeared only in the training set (white) or the testing set (gray). For example, subjects 1, 2, and 3 contributed to the training sets of folds 1 to 9, but were excluded from the training set of fold 10, in which subjects 1, 2, and 3 were part of the test set. Therefore, overall, our assessment of the model’s performance captured its ability to generalize across subjects.

The same duration criterion (i.e., 10 seconds) was applied to all SHHS data irrespective of the apnea type; physiological criteria were used to distinguish the apnea types (Fig 1). Because the duration criterion for UCD was unknown, we applied the SHHS criterion. Therefore, for SHHS and UCD data sets, we first divided the data into 30-second segments and then labelled all segments that contained at least 10 continuous seconds of “obstructive sleep apnea”, “central sleep apnea”, “mixed apnea”, or “hypopnea” as, simply, “apnea”. All other segments were labelled “non-apnea”. For the MIT data set, we used the labels provided. All three data sets scored sleep stages using the Rechtshaffen and Kales criteria [38], albeit the SHHS dataset used a modified version of these criteria [35]. Channel C4-A1 [39] was the only channel present across all three datasets and therefore was used exclusively to ensure consistency.

### Classifier design

**Fig 2:**
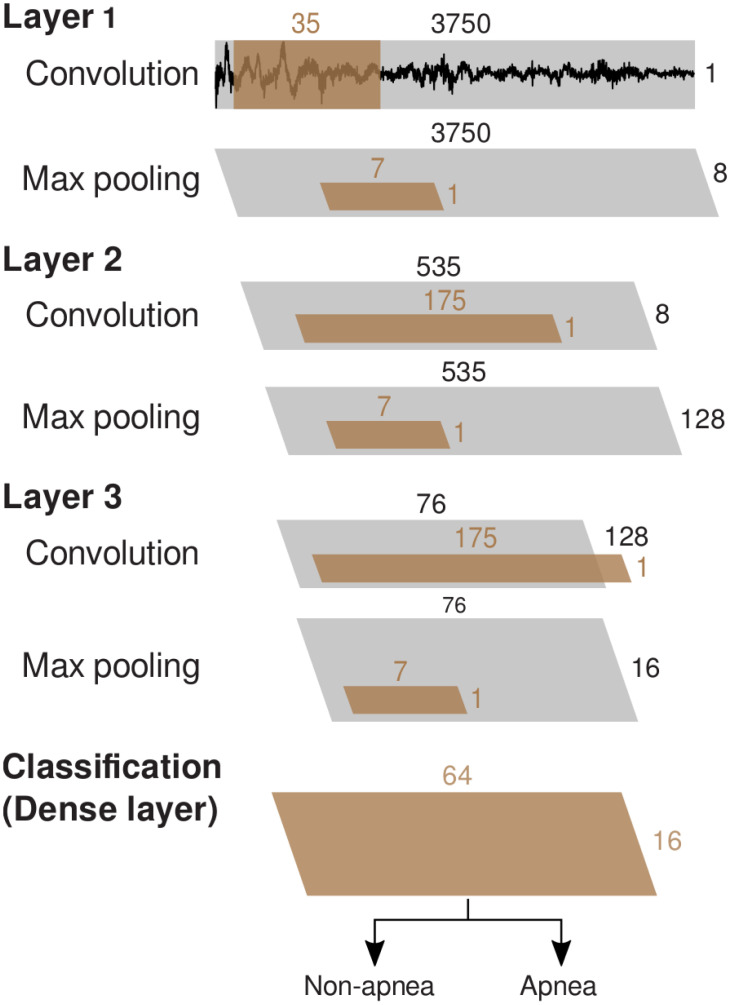
The architecture of our CNN trained to detect SA, comprising three convolutional layers. Convolutions had a stride size of one and used zero padding. Each convolutional layer was followed by batch normalisation, ELU activation, and dropout (these operations are not illustrated). The dense layer was preceded by a flattening operation, and followed by ELU activation and a dropout layer. The output layer used a softmax classifier [42]. (Symbology after Schirrmeister and colleagues [27]).

Schirrmeister and colleagues [27], evaluated shallow, deep, hybrid and residual convolutional neural networks, as well as the filter bank common spatial patterns (FBCSP) algorithm. These algorithms were evaluated across several multi-channel EEG motor-decoding datasets. They concluded that shallow and deep architectures reached, and sometimes exceeded the performance of FBCSP (the de facto standard), in contrast to hybrid and residual neural networks which fell short of the performance of FBCSP. In light of their findings, we constructed our CNN network (Fig 2) using three convolutional layers; each convolutional layer involved normalization, exponential linear unit (ELU) activation, and max-pooling. We kept our network architecture simple with a relatively low number of convolutional layers, as we anticipated that it would better facilitate explainability (i.e., mechanistic analysis of the trained network’s performance, described below). We used batch normalisation (after each convolution layer) and dropout (after each pooling layer) [40] to improve network stability and safeguard against overfitting. As illustrated in Fig 2, the last convolutional layer was followed by a dense layer, and an output layer with the softmax activation function. We initialized the weights of our CNN by drawing from a truncated normal distribution with zero mean. To train the CNN, we minimized the cross-entropy loss function through backpropagation. Backpropagation was optimized using Adam [41] in the standard fashion: alpha coefficient was set at 0.9, and beta coefficient at 0.999. The learning rate was tuned alongside other hyperparameters (see Table 1).

**Table 1:** Optimized hyperparameters, and hyperparameter search spaces. The rightmost column shows optimal hyperparameters evaluated with Hyperband-based tuning.

Layer	Hyperparameter	Search space	Final
Convolutional layer 1	Kernel length	25,35,50,75,125,175	35
	Number of filters	8,16,32,64,128	8
Convolutional layer 2	Kernel length	25,35,50,75,125,175	175
	Number of filters	8,16,32,64,128	128
Convolutional layer 3	Kernel length	25,35,50,75,125,175	175
	Number of filters	8,16,32,64,128	16
MaxPooling layer	Window/stride size	3,5,7,9	7
Dense layer	Number of nodes	16,32,64,128,256	64
Convolutional layer dropout	Dropout rate	0 to 0.6	0.1
Dense layer dropout	Dropout rate	0 to 0.6	0.0
Optimizer	Learning rate	0.0001 to 0.1	0.00163

### Hyperparameter tuning

There are few studies describing how best to optimize network hyperparameters (i.e. network variables set prior to training) to detect SA in EEG. Therefore we approached hyperparameter optimization in a semi-automated manner. First, we reviewed literature that reported hyperparameter values, and we constructed a hyperparameter search space encompassing the values we found. Second, we systematically searched this space for optimal hyperparameter combinations. We considered the following search strategies: random-search, as well as Bayesian-based [43] and Hyperband-based [44] algorithms. In preliminary experiments, we tested all approaches, finding that the Hyperband-based approach outperformed the others. The search space and the selected hyperparameters are listed in Table 1. We adjusted Hyperband such that the algorithm was repeated five times, with one-third of hyperparameter configurations kept during each successive halving sub-operation. To make hyperparameter optimization computationally tractable, some hyperparameters (e.g., number of convolutional layers and activation function) were selected based on previous work, specifically Schirrmeister and colleagues [27].

### Evaluation process

For our network, we performed subjectwise 10-fold cross-validation to assess performance. For each of these 10 folds, subjects were allocated to either the training set (approx. 81%), the testing set (approx. 10%), or the validation set (approx. 9%). The training and validation sets were randomly undersampled based on the minority class for each subject’s recording. Undersampling was not performed on the testing set (Table 2). We reasoned that training on highly unbalanced data is undesirable, as the classifier may develop bias towards the majority class (i.e., the non-apnea class). Network training was performed for 40 epochs (i.e., 40 passes of the training data through the network) with Python 3.7 and Tensorflow 2.2, on NeSI (New Zealand eScience Infrastructure), a high-performance computing platform which uses Tesla P100 GPU cards (NVIDIA, Santa Clara, California, United States). Computation took on average 112 microseconds per input sample for training and 40 microseconds per sample for testing. Furthermore, to evaluate our CNN’s ability to generalize across datasets, we tested its performance (after training it using the SHHS dataset) on the UCD and MIT-BIH datasets described above.

**Table 2:** The mean distribution of annotations within the training and testing sets. The validation set has the same proportions as the training set. Each EEG segment has an apnea annotation (i.e., “apnea” or “non-apnea”) and a sleep-stage annotation (i.e., “wake”, “REM” or “NREM”). Overall there was on average 1,144 segments per patient before undersampling and 378 segments per patient after resampling.

Number of annotations	Apnea	Non-apnea	Wake	REM	NREM
Training	407625.6	407625.6	202858.8	174796.6	437595.8
Testing	250424.2	50461.9	118989.6	35089.1	146807.4

To quantify performance of our trained CNN, we used accuracy and Matthews correlation coefficient (MCC). Accuracy is a common metric used to evaluate neural network performance. However, accuracy may be susceptible to biases when data sets are unbalanced (i.e., data sets containing a preponderance of one or other class labels). Therefore, we also calculated MCC—a robust performance measure suitable for unbalanced testing data sets [45, 46]. Additionally, we developed a shuffle test to conservatively estimate performance baselines (referred to hereafter as the “conservative baseline”). To estimate baselines, we shuffled the class labels in the training set and performed training and testing using 10-fold subjectwise cross-validation; the measured performance provided our baselines. We reasoned that a CNN trained on these shuffled labels would be incapable of learning salient EEG features for SA detection, but could nonetheless learn the statistics of data set imbalance, and bias its behaviour accordingly. We used a Bayesian t-test, computing 95% highest density intervals (HDIs) [47], to compare our CNN’s performance to baseline.

### Critical-band masking

To explain the mechanisms of our trained networks, we used a critical-band masking (CBM) technique that was adapted from psychoacoustics [31] and visual psychophysics [32]. Here, we added bandlimited noise to test segments (but not training segments). We used a noise bandwidth of 1.5 Hz and parametrically varied the noise frequency centred from 1.5 Hz to 60 Hz in increments of 1.5 Hz. A finite-impulse-response (FIR) band-pass filter was applied to white noise to create this bandlimited noise (length = 825, transitional bandwidth = 0.5 Hz). At each center frequency, we quantified noise intensity by computing the log of the noise root-mean-square (RMS) value and signal RMS value to find the signal-to-noise ratio (SNR). For each band center frequency, we tested the trained network using these noisy test segments, quantifying the deleterious effect of noise by observing changes in MCC scores. This CBM process was performed for every fold of our subjectwise 10-fold cross-validation (see Evaluation Process).

### Filter lesioning

We adapted a “lesioning” technique from Lawhern et al. [33] to determine the relative importance of first-layer convolutional kernels to the trained network. On each fold, after having trained the network, we zeroed all coefficients for one convolutional kernel, and then tested the network. We therefore quantified the deleterious effect that zeroing (i.e., lesioning) kernels had on test performance. We did this for each first-layer convolutional kernel on each fold of our subjectwise 10-fold cross-validation. Thus, on each fold, we were able to rank 1st-layer convolutional kernels by importance; e.g., the most important kernel, when lesioned, caused the greatest reduction in network performance. To verify that this lesioning technique was effective in identifying important convolutional kernels, we computed a correlation coefficient between all pairs of kernels within and between folds; specifically, we computed Pearson’s correlation coefficient between kernels’ Fourier amplitude spectra. The correlation coefficient was generally higher between convolutional kernels deemed to be important, both within and between folds. Finally, we computed the Fourier transform, and calculated the z-scores of the most important kernels (i.e., those determined important by Filter Lesioning). To do so, we formed a null distribution of kernel transforms using all trained kernels across all folds.

## Results

**Fig 3:**
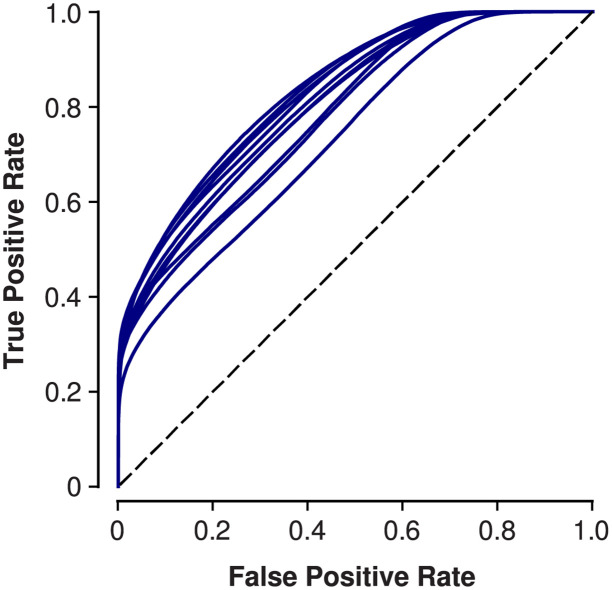
ROC curves summarizing the performance of our SA network. We represent each fold of our subjectwise 10-fold cross-validation with a separate curve. Across folds, the AUC averaged 0.804 (s.t.d. = 0.031).

Our network detected SA with an accuracy equal to 69.9% (the mean across folds of our subjectwise 10-fold cross-validation). The standard deviation (s.t.d.) of this accuracy was 3.0 percentage points across folds. Our network performed with an MCC score equal to 0.375 (s.t.d. = 0.017). The MCC performance metric was reliably above the conservative baseline (difference in MCC: Bayesian t-test, 0.374, 100% HDI; Methods); baseline MCC was 0.00 (s.t.d. = 0.04). Baseline accuracy was 49.9% (s.t.d. = 32.4 percentage points). After training with shuffled labels, the network tended to guess, from one fold to the next, either all apnea or all non-apnea. This resulted in a bimodal distribution for baseline accuracy, which we therefore did not use with a Bayesian t-test for statistical inference. In addition to measuring accuracy and MCC, we also undertook a receiver operating characteristic (ROC) analysis of our network’s performance (Fig 3). Averaged across folds, the area under the ROC curve (AUC) was equal to 0.804 (s.t.d. = 0.031).

We then tested this trained network using data drawn from other datasets. Our network predicted the MIT dataset with a MCC equal to 0.216 (s.t.d. = 0.125). These performance metrics were reliably above our conservative baseline with a probability of 95.6% (difference in MCC: Bayesian t-test, 0.201); baseline MCC was -0.03 (s.t.d. = 0.18). Our network predicted the UCD dataset with a MCC equal to 0.169 (s.t.d. = 0.120). The performance metrics after testing on the UCD dataset were reliably above our conservative baseline (difference in MCC: Bayesian t-test, 0.157, 100% HDI; Methods); baseline MCC was 0.025 (s.t.d. = 0.09).

**Fig 4:**
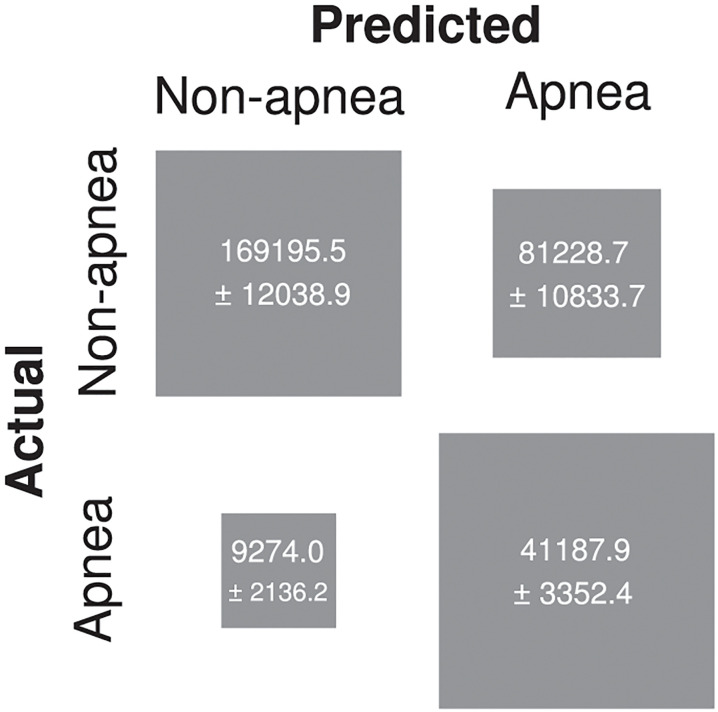
Confusion matrix, summarizing the performance of our SA network. The area of each square represents the value of each matrix entry. Values are counts averaged across our subjectwise 10-fold cross-validation. The intervals (±) associated with each value show s.t.d. across folds. Overall, the network performed with accuracy = 76.8%, as indicated by the mass along the matrix’s main diagonal.

**Fig 5:**
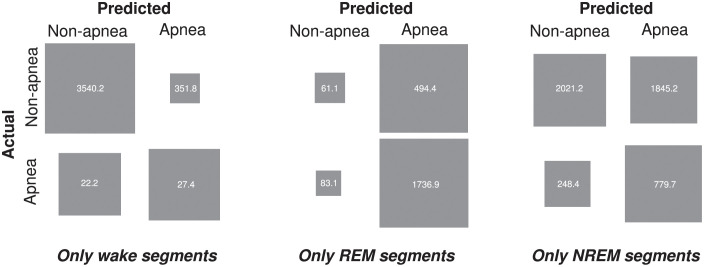
Confusion submatrices, each corresponding to one or other of three sleep stages: Wake (left), REM (middle), and NREM (right). For wake and REM, our SA network appeared to behave in a biased fashion. Graphical conventions are as in Fig 4.

Several previous studies have found that SA is associated with sleep stage (a point we elaborate in Discussion). Therefore, we wondered if our trained SA network, to aid its classification, was representing sleep stage-associated features (i.e., covertly decoding sleep stage). To examine this idea, we decomposed the confusion matrix (Fig 4) into three submatrices, each corresponding to one of three sleep stages (Fig 5): wake, rapid-eye-movement (REM), and non-rapid-eye-movement (NREM). Upon inspection, parts of these submatrices appeared to indicate our SA network behaved in a biassed fashion. For instance, segments recorded during REM sleep predicted apnea on 93% of all trials. This potentially indicates that our trained SA network was representing (i.e., covertly decoding) sleep stage and, since SA is associated with sleep stage, used these representations to aid its performance in SA detection.

To test this hypothesis—that our CNN was representing sleep stage—we adapted our shuffle test (see Evaluation Procedure) to account for sleep stage; training data were split according to three sleep stages (wake, REM, NREM) and, for each stage, the labels “apnea” and “non-apnea” were shuffled prior to re-training the network (below, we refer to this procedure as re-training the network after a “stage-wise shuffle” of the data). We reasoned that, if our SA network was, in fact, representing sleep stage to aid its detection of SA, this stage-wise shuffle would isolate the effect of this representation on SA detection performance. Specifically, if, after we re-trained the network using stage-wise shuffled data, network performance was unaltered, then that would indicate that the representation of sleep stage was wholly responsible for SA detection. On the other hand, if, after we re-trained the network using stage-wise shuffled data, network performance fell to baseline, then that would indicate that the representation of sleep stage was not being used to aid SA detection. After re-training the network on stage-wise shuffled data, the network performed with an accuracy equal to 56.7% (the mean across folds of our subjectwise 10-fold cross-validation; s.t.d. = 4.1 percentage points across folds), and a MCC score equal to 0.275 (s.t.d. = 0.019). The MCC was reliably below that of the original SA network (difference MCC: Bayesian t-test, -0.099, 100% HDI; Methods). Therefore, our SA network appeared to learn a representation of sleep stage to aid its detection of SA, but the network’s learning of this representation only partly accounted for its ability to detect SA.

To explore further this idea—that our SA network was representing sleep stages—we developed a second CNN. This second CNN was nearly identical to our original SA network (the one exception being that the second CNN had five nodes in the final dense layer as opposed to two); we trained this network to decode sleep stages (wake, REM, and NREM). This sleep-stage network performed with an accuracy equal to 85.3% (mean across folds of our subjectwise 10-fold cross-validation; s.t.d. = 0.57 percentage points across folds), and a MCC score equal to 0.766 (s.t.d. = 0.009 across folds). The MCC was reliably below that of the original SA network (difference in MCC: Bayesian t-test, 0.766, 100% HDI; Methods); baseline accuracy for our sleep stage network was 52.0% (s.t.d. = 0.54 percentage points), and baseline MCC was 0.000 (s.t.d. = 0.001). Therefore, a network with architecture nearly identical to that of our SA network can be trained, explicitly, to decode sleep stage. This adds further support to our idea that our SA network learnt to represent (i.e., covertly decode) sleep stage, and it used this representation to aid in the detection of SA.

**Fig 6:**
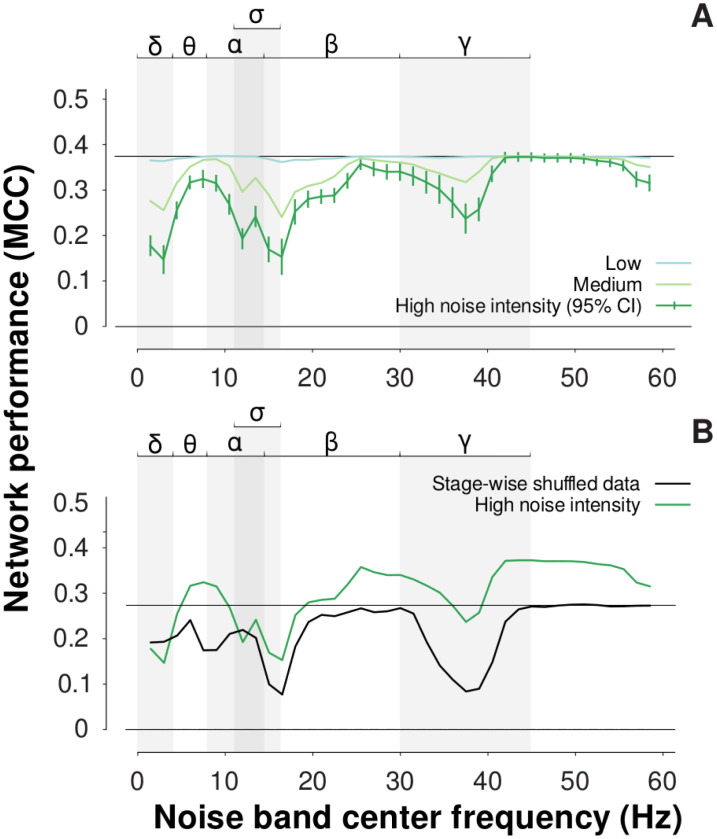
(A) Effect of critical-band masking on our SA network’s performance. We used high-, medium-, and low-intensity noise: SNR = 5, 10, and 20, respectively (Methods). Overall, high-intensity noise decreased performance more than low-intensity noise. The deleterious effect of noise was pronounced in some frequency bands but not others. E.g., adding bandlimited noise to test signals in the delta band (< 4 Hz) caused MCC to decrease from 0.38 to 0.14. The lower, horizontal solid line (performance = 0.0 MCC) indicates the performance baseline (Methods), and the upper, horizontal solid line indicates network performance in the absence of noise (MCC = 0.38). The error bars (shown only for high-intensity noise) are 95% confidence intervals computed across folds of our subjectwise 10-fold cross-validation. The Greek letters (top) mark traditional frequency bands; sigma marks the band associated with sleep spindles. (B) Effect of critical-band masking on our re-trained SA network; we re-trained the network after data were stage-wise shuffled. Adding bandlimited noise to test signals in the delta and alpha (8 to 13 Hz) bands, here, had little effect on network performance. By contrast, noise in the lower beta band and gamma band heavily reduced performance. Other graphical conventions are as in (A). Upper horizontal line marks the stage-wise shuffled no-noise response (MCC = 0.275).

We wondered what EEG features were used by our SA network in performing SA detection. Therefore, we used critical-band masking (CBM), adding bandlimited noise to the signals used to test our SA network (Methods). The effects of CBM were graded; for the addition of low-intensity noise (SNR = 20; Methods), masking had little effect on network performance, regardless of the noise band’s center frequency. When we increased the intensity of noise, network performance deteriorated (i.e., MCC decreased). Deterioration was pronounced for noise in some frequency bands but not others. Overall, the effects of CBM were primarily limited to three regions (Fig 6A): frequencies less than 4 Hz (the delta band); 30 to 45 Hz (the gamma band); and frequencies running from approximately 10 to 20 Hz, encompassing alpha (8 to 13 Hz), sleep spindles (11 to 16 Hz), and the lower beta band (14 to 30 Hz). Notably, when high-intensity (SNR = 0) noise was added to the delta band, MCC for SA detection was reduced from approximately 0.38 to 0.14. When high-intensity noise was added to the band associated with sleep spindles (11 to 16 Hz), MCC for SA detection was reduced from approximately 0.38 to 0.16. When high-intensity noise was added to the gamma band, MCC for SA detection was reduced from approximately 0.38 to a minimum of 0.22.

For comparison, we also applied CBM to the network that we re-trained using stage-wise shuffled data (see above). We reasoned that the different effects of CBM on those two networks (our original SA network, and the network re-trained on stage-wise shuffled data) would help determine which frequency bands were important to SA detection per se, which were important to sleep stage decoding (which appears to play a role in SA detection), and which were important to both. A key outcome of this experiment involved the delta band (Fig 6B); while noise in the delta band caused deterioration in the performance of our original SA network, it had relatively little effect on the network re-trained on stage-wise shuffled data. This difference indicated that, while other frequency bands may have contributed to the network’s representation of sleep stage (e.g., sleep spindles, 11 to 16 Hz), delta band activity was specifically important to SA detection. This result is consistent with several studies that have observed an association between SA events during sleep and delta-band activity [27, 34] (a point elaborated in Discussion).

**Fig 7:**
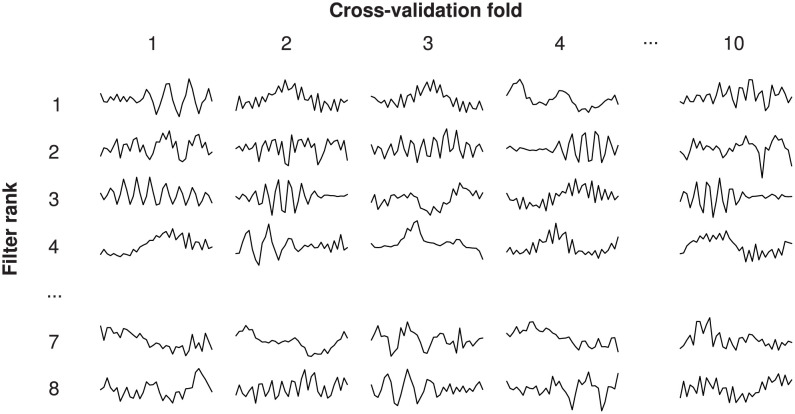
Importance matrix, showing 1st-layer filters comprising our trained SA network. Matrix columns correspond to folds from our subjectwise 10-fold cross-validation; rows correspond to importance (i.e., the most important filter on each fold is shown in row 1). To illustrate by example, on the first fold of cross-validation, the filter kernel illustrated at column 1, row 1 (top-left), was determined to be the most important; lesioning this filter reduced the trained SA network’s performance more significantly than any other filter on this fold.

**Fig 8:**
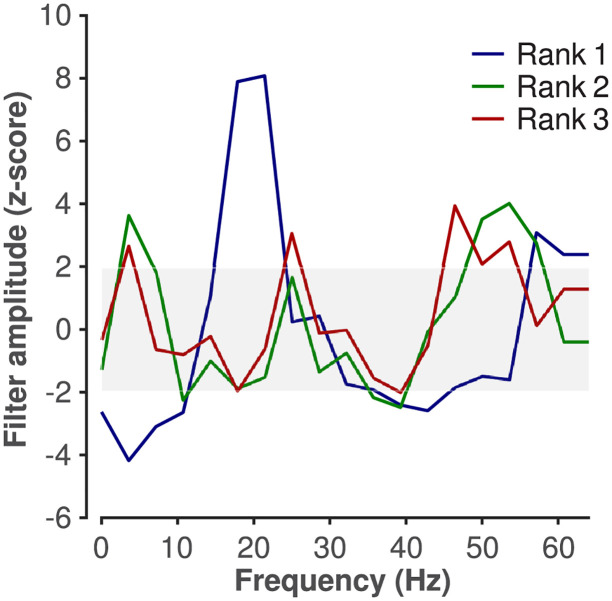
Amplitude spectra of 1st-layer filters important to the SA network’s performance. We show spectra for the 1st-, 2nd-, and 3rd-most important filters (“Rank 1, 2, and 3”, respectively). Important filters appeared to attenuate the delta band, and amplify the beta and gamma bands. The shaded rectangle marks a 95%-confidence interval (i.e., -1.96 < z-score < 1.96), wherein the spectral amplitude of rank 1, 2, and 3 filters is not appreciably different from that of all other 1st-layer filters comprising the ensemble.

Critical band masking indicated that specific frequency bands were important for SA detection, especially the delta band. We therefore wondered whether filters in the first convolutional layer of our trained SA network responded selectively to EEG signals in these bands. To answer this question, we lesioned first-layer filters comprising the trained SA network (Methods). We found that, when lesioned, some filters substantially reduced the performance of our SA network (i.e., these filters appeared important to the network). The lesioning of other filters, by comparison, caused negligible deleterious effects (i.e., apparently unimportant filters). We sorted 1st-layer filters based on importance. These sorted filters were then used to generate an “importance matrix” (Fig 7). In light of that importance matrix we asked, “Do important filters have similar characteristics?” To answer this question, we computed each 1st-layer filter’s amplitude spectrum using the Fourier transform, and we averaged spectra within importance and across folds (i.e., after Fourier transformation, we averaged amplitude spectra within rows, across columns, of Fig 7). When we compared the amplitude spectra of relatively important filters to the ensemble (i.e., all filters comprising Fig 7), we found the following (Fig 8): important 1st-layer filters tended to attenuate the delta band, and amplify the beta and gamma bands. This pattern is consistent with the result of our critical-band masking.

## Discussion

We have developed a CNN that detects SA from single-channel EEG (accuracy = 69.9%; baseline = 49.9%). Our CNN’s performance, when measured using MCC (MCC = 0.38; baseline = 0.03), showed a “weak-moderate positive correlation” [48] (conventionally, MCC is interpreted in a similar fashion to Pearson’s correlation coefficient [48]). Furthermore, our network’s performance was robust when measured using ROC analysis (across folds, average AUC = 0.804). To explain the mechanisms of our CNN, we used two techniques—critical-band masking (wherein band-limited noise was added to signals used to test the trained network) and filter lesioning (wherein 1st-layer filter kernels were zeroed in turn). To our knowledge, the use of critical band masking is a novel approach to analysing a trained CNN. Our results indicate all three of our hypotheses, which we outlined in the Introduction, were confirmed: there is information in single-channel EEG enabling reliable subjectwise detection of SA by an explainable CNN; knowledge of sleep stage appeared to improve SA detection; and, our CNN used information contained in single-channel EEG that is consistent with known SA biomarkers, specifically the delta and beta bands.

Previous work has demonstrated that SA is accompanied by characteristic alterations of EEG [49, 50]. Likewise, the sleep stages—wake, REM, and the three NREM stages (N1, N2, N3)—all also are accompanied by systematic changes in EEG [51, 52]. Azim and colleagues [50] studied the normalized Welch power spectral density of electrode C4-A1 from the UCD database [36]. They found that during SA events, power in the beta band (i.e., frequencies between 14 and 30 Hz) decreased (compared to pre-apnea events), before rising again after SA event termination. During different stages of sleep, the EEG features associated with SA can change. For example, apneas occurring during NREM sleep are associated with a gradual increase in delta-band activity (i.e., at frequencies < 4 Hz), followed by a decrease within that band concomitant with patient arousal and/or wakefulness [49, 50]. In contrast, apneas occurring during REM sleep are associated with transient increases in delta-band activity [49, 50]. REM SA events are also generally associated with small increases to beta band activities. Physiological differences between sleep stages can also impact SA. REM sleep causes the relaxation of muscle tone which is conducive to SA events [53]. Therefore, EEG—a signal which contains characteristics for both SA and sleep stage—should in theory be useful for detecting SA.

Our use of critical-band masking and filter lesioning, taken together, indicated that our SA network learned to rely on known SA as well as sleep stage biomarkers. Critical-band masking indicated that delta-band activity was important to the detection of SA; that beta- and gamma-band activity was important to the decoding of sleep stage; and that alpha-band activity may have played a role in both SA detection and sleep stage decoding. We made these inferences by adding noise, first, to signals used to test our trained SA network, and, second, to signals used to test the network after re-training with stage-wise shuffled data. Our masking results were in broad agreement with our lesioning results. The trained SA network’s most important 1st-layer filters selectively attenuated delta-band activity, and selectively amplified activity between 14 and 30 Hz (i.e., the beta band). Taken together, our findings are broadly consistent with existing work; it has been previously shown using spectral analysis of multi-channel EEG recorded from SA sufferers that delta-band activity is associated with SA events [49, 50]. Furthermore, REM and NREM sleep are associated with the reduction of power in beta- and gamma bands [54].

The present study is subject to two main limitations, the first of which concerns the generalisation of our CNN across datasets. We trained our CNN using the SHHS dataset; subjectwise 10-fold cross-validation using test data from SHHS (ie., within-dataset testing) showed good performance (see Results). However, when we tested our SHHS-trained CNN using data from the UCD or MIT-BIH datasets (ie., across-dataset testing), its performance was somewhat sobering (see Results). This performance reduction is possibly attributable to low inter-rater reliability (IRR). For the SHHS dataset, the three main SA scorers were in only moderate agreement (scorers 914 and 915, Cohen’s kappa = 0.7; scorers 914 and 916, 0.73; scorers 915 and 916, 0.76) [55]. Estimates of IRR between datasets (e.g., between SHHS and UCD) is presently unavailable, however it stands to reason that it is significantly lower than 0.7 because SA annotation procedures differed considerably between datasets (see Methods). The precise relationship between IRR and the theoretical maximum performance of our CNN, and the way in which changes to IRR in turn affect CNN performance, is a subject of our ongoing research. The second limitation of our study concerns labelling; we labelled segments used in the training, testing, and validation of our CNN as either “apnea” or “non-apnea” despite the fact that there are several clinical SA subtypes, including obstructive, central, and mixed SA, and hypopnea [56]. We grouped these subtypes for two reasons. First, there were relatively few instances of central SA in these datasets; had we attempted to balance our training set, for each subject, with regard to subtype, there would have been too few data to train a reliable CNN. Second, IRR is low as regards discriminating obstructive from mixed SA; in the SHHS dataset, these subtypes are grouped under the label “obstructive SA”. The development of an architecture capable of discriminating between SA subtypes is a subject of our ongoing research.

To our knowledge, Jiang and colleagues [57] is the only other group to develop a CNN for SA classification using single-channel EEG. We have extended their work in several ways, albeit comparison between their results and ours, for reasons outlined below, is not straightforward. First, Jiang et al. used a small dataset (the MIT-BIH Polysomnographic Database [37]), comprising recordings from only 16 participants. By contrast, our study integrated recordings from three databases, comprising over 2600 participants. Second, Jiang et al. appear to have used an unbalanced data set to train and test their network (specifically, their dataset appears to have overrepresented apnea, as opposed to non-apnea, EEG recordings); in general, the use of unbalanced data sets may bias estimates of a classifier’s performance. By contrast, we were careful to balance data before training our CNN, and we developed a shuffle test to ensure that our estimates of baseline performance were conservative. Third, Jiang et al. performed pooled (not subjectwise) cross-validation. By “pooled”, we mean that on each fold of their cross-validation procedure, recordings from all subjects were contained in both training and testing sets. Because they pooled data, it is unclear whether their results can generalize in a clinical setting (i.e., it would be clinically useful if a CNN, trained using data recorded from a normative cohort of subjects 1 through N, could be used to detect SA in recordings from a previously unseen subject N+1). By contrast, we used subjectwise cross-validation, which indicates that our results will generalize to unseen participants, and therefore may have clinical potential for diagnosis. Fourth, the network of Jiang et al. was, by contrast to ours, rather complex, and therefore ill-suited for explanation; the network employed a hybrid architecture with 4 parallel, computational branches: two shallow branches (each of which comprised three convolutional layers), and two deep branches (comprising 6 and 9 convolutional layers, respectively). By contrast, our network was simple, and this simplicity enabled an explanation of its function. It is therefore our expectation that our explainable CNN has clinical potential for the improved detection, diagnosis, and treatment of SA.

## Conclusion

EEG offers an alternative way to detect SA, and detection appears to benefit from sleep stage information contained in EEG. Single-channel EEG is low-instrumentation, making it potentially suitable for in-home sleep and SA monitoring. Here, we have developed a CNN that reliably detects SA from single-channel EEG. Our visualization techniques indicated that the classifier learned frequency-band information consistent with known SA biomarkers. It is a priority for future work to quantify our algorithm on consumer-grade EEG equipment.
